# PrEP as a feature in the optimal landscape of combination HIV prevention in sub-Saharan Africa

**DOI:** 10.7448/IAS.19.7.21104

**Published:** 2016-10-18

**Authors:** Jessica B McGillen, Sarah-Jane Anderson, Timothy B Hallett

**Affiliations:** HIV Modelling Consortium, Department of Infectious Disease Epidemiology, Imperial College London, London, UK

**Keywords:** PrEP, combination prevention, optimisation, sub-Saharan Africa

## Abstract

**Introduction:**

The new WHO guidelines recommend offering pre-exposure prophylaxis (PrEP) to people who are at substantial risk of HIV infection. However, where PrEP should be prioritised, and for which population groups, remains an open question. The HIV landscape in sub-Saharan Africa features limited prevention resources, multiple options for achieving cost saving, and epidemic heterogeneity. This paper examines what role PrEP should play in optimal prevention in this complex and dynamic landscape.

**Methods:**

We use a model that was previously developed to capture subnational HIV transmission in sub-Saharan Africa. With this model, we can consider how prevention funds could be distributed across and within countries throughout sub-Saharan Africa to enable optimal HIV prevention (that is, avert the greatest number of infections for the lowest cost). Here, we focus on PrEP to elucidate where, and to whom, it would optimally be offered in portfolios of interventions (alongside voluntary medical male circumcision, treatment as prevention, and behaviour change communication). Over a range of continental expenditure levels, we use our model to explore prevention patterns that incorporate PrEP, exclude PrEP, or implement PrEP according to a fixed incidence threshold.

**Results:**

At low-to-moderate levels of total prevention expenditure, we find that the optimal intervention portfolios would include PrEP in only a few regions and primarily for female sex workers (FSW). Prioritisation of PrEP would expand with increasing total expenditure, such that the optimal prevention portfolios would offer PrEP in more subnational regions and increasingly for men who have sex with men (MSM) and the lower incidence general population. The marginal benefit of including PrEP among the available interventions increases with overall expenditure by up to 14% (relative to excluding PrEP). The minimum baseline incidence for the optimal offer of PrEP declines for all population groups as expenditure increases. We find that using a fixed incidence benchmark to guide PrEP decisions would incur considerable losses in impact (up to 7%) compared with an approach that uses PrEP more flexibly in light of prevailing budget conditions.

**Conclusions:**

Our findings suggest that, for an optimal distribution of prevention resources, choices of whether to implement PrEP in subnational regions should depend on the scope for impact of other possible interventions, local incidence in population groups, and total resources available. If prevention funding were to become restricted in the future, it may be suboptimal to use PrEP according to a fixed incidence benchmark, and other prevention modalities may be more cost-effective. In contrast, expansions in funding could permit PrEP to be used to its full potential in epidemiologically driven prevention portfolios and thereby enable a more cost-effective HIV response across Africa.

## Introduction

The long-term provision of HIV treatment presents a financial burden that is likely to approach the sum total of the national debt of some affected countries [1]. Scaling up combination HIV prevention is widely recognised as vital for ensuring progress against the epidemic and alleviating this economic burden on low- and middle-income countries. Clinical trials have shown oral pre-exposure prophylaxis (PrEP) to be an effective method for preventing HIV transmission [2–7], with protection and adherence strongly correlated [8]. Ahead of results from these trials, the early PrEP literature laid the groundwork for considering the future role of PrEP and how its introduction was expected to change the prevention landscape [9,10]. More recently, practical issues of implementation in resource-limited countries have been discussed [11–13], and the importance of determining how best to position PrEP within combination prevention efforts has been emphasised [13–15].

The WHO guidelines now recommend offering PrEP to people who are at substantial risk of HIV infection [16]. What this looks like at scale, in terms of where and how PrEP should be implemented, comprises an open question of immediate importance for shaping our progress toward ambitious global goals [17]. This is particularly true for sub-Saharan Africa, where the burden of disease is highest in the world [18,19] and resources available for the HIV response are likely to remain limited [20,21]. There is thus considerable scope for mathematical modelling to confront the complexity of the HIV epidemic in sub-Saharan Africa and inform our approach to PrEP implementation there.

A recent modelling study [22] evaluated the potential of a five-year PrEP intervention targeting the general adult population in sub-Saharan Africa. PrEP was found to be cost-effective in countries with high HIV burdens and low rates of male circumcision, though this was against a backdrop of homogeneous sexual behaviour and fixed national coverage levels for other interventions. Anderson *et al*. [23] included PrEP in the first rigorous comparison of the cost-effectiveness of intervention portfolios across heterogeneous subnational regions, with the scope of Kenya. We recently reported how funds for combination prevention could be rebalanced within and across multiple countries for a more effective continent-wide response against HIV [24]. PrEP emerged as an important prevention modality in this analysis, with a role in the optimal prevention strategy that is complex and merits closer examination.

Here, we use the optimal prevention strategy from our previous work [24] as a springboard to carry out an analysis of where, and to whom, PrEP would ideally be offered in the resource-limited, heterogeneous setting of sub-Saharan Africa. Our study serves as a demonstration of the principle that widening the context in which an intervention is evaluated can increase both the effectiveness of that intervention and the cumulative impact of combination prevention.

## Methods

### Model structure and prevention optimisation approach

Full details of the model structure used here, its parameters, and their calibration to data can be found in the recent paper by McGillen *et al*. [24]. For brevity, we restrict our description to a summary of key features. At the centre of this framework is a dynamic compartmental model that describes sexual HIV transmission, deaths, and prevention. The model is used to describe each top-level administrative subnational region in 18 countries (Benin, Botswana, Burkina Faso, Cameroon, Congo, Democratic Republic of the Congo, Ethiopia, Kenya, Mali, Mozambique, Nigeria, Rwanda, Sierra Leone, South Africa, Swaziland, Tanzania, Zambia, and Zimbabwe), in total capturing 80% of the burden by people living with HIV in sub-Saharan Africa.

In each subnational region, the population is grouped by risk level. Key high-risk populations are female sex workers (FSW) and men who have sex with men (MSM). The general population includes low-risk women and men, who tend to form stable long-term partnerships; moderate-risk women and men, who form casual partnerships; and high-risk male clients of sex workers. Heterosexual HIV transmission links men and women, homosexual HIV transmission links MSM, and risk structure further differentiates how the population groups interact and how HIV is transmitted among and between them. Parameters representing sexual behaviours and sexual mixing patterns comprise the proximate determinants of risk and can vary between these groups. After HIV infection, the model tracks disease progression by CD4 status [25].

In [24], parameters governing biological aspects of HIV (such as disease progression rates) were held constant across subnational regions. For each subnational region, parameters governing sexual behaviours, local epidemiological characteristics, population demographics, and historical treatment and prevention initiatives were incorporated into a standard likelihood expression, which was maximised using a direct-search simplex algorithm. This produced epidemic dynamics consistent with local data on prevalence levels [26–28], prevalence trends [29], historical circumcision levels [26], demographic characteristics of the general population [30,31] and high-risk population groups [32–50], and historical scale-up of antiretroviral therapy (ART) coverage [51]. The calibration process was repeated for all subnational regions (detailed fully in [24]).

Although detailed data on sexual behaviours among MSM are available for some of Kenya's major urban centres [52,53] and coastal districts [54,55], these do not necessarily generalise to other locations in sub-Saharan Africa, and data are otherwise extremely scarce. This limits the sophistication of our representation of MSM. However, viral genotyping data indicate that transmission between MSM is not isolated from transmission in the general population [56], and studies from across Africa suggest that 41% to 86% of MSM have also had sex with women in their lifetime [50]. We thus assumed that all MSM can form partnerships with both sexes, and allowed the ratio of male to female partnerships to vary among subnational regions between a value of 1 (no preference for male partnerships) and 20 (strong preference for male partnerships).

Prevention interventions can be targeted to different population groups to change their proximate determinants of risk and thereby affect the cumulative incidence across population groups. Prevention interventions considered in [24] are oral PrEP, behaviour change communication, voluntary medical male circumcision, and outreach testing with the offer of ART to all HIV-positive people, which we call “early” ART as it typically reaches people who are early in disease progression and would otherwise be unlikely to present for care. In the model, PrEP and voluntary medical male circumcision reduce the per-partnership probability of HIV acquisition, and behaviour change communication reduces the mean rate of changing partners. Early ART amplifies the number of HIV-positive people on ART, reducing onward transmission. PrEP and behaviour change communication can be offered to HIV-negative people, voluntary medical male circumcision to uncircumcised HIV-negative men, and early ART to all HIV-positive people who have not already presented for care.

We assigned unit costs to these prevention modalities in accordance with the UNAIDS Fast Track framework [17] and made a set of assumptions around achievable maximum coverage levels in the population groups (Table 1). Current coverage levels vary by subnational region for behaviour change communication and voluntary medical male circumcision, depending on historical initiatives, while early ART and PrEP are assumed to start from the beginning of the 15-year intervention period (2016 to 2030). In the absence of prevention scale-up, we assume that the effects of past behaviour change and circumcision campaigns would persist, but early ART and PrEP would not be introduced. In assuming a maximum possible scaled-up PrEP coverage of 50% for the key populations, our model is slightly more ambitious than the UNAIDS Fast Track, which reports an assumption of “PrEP for 30% (2030) of key populations” [17]. We further assume that PrEP provides a 75% reduction in the risk of HIV acquisition (Table 1). This effectiveness level accommodates imperfect adherence and accords with PrEP trials that have found risk reductions ranging from 42% (whole study arm) to 92% to 99% (good adherers) among gay and bisexual men and transwomen [2] and from 62% (whole study arm) [3] to more than 90% (good adherers) [4] among heterosexual men and women.

**Table 1:** Prevention interventions

Intervention	Target population	Effectiveness	Unit cost	Achievable coverage (%)
VMMC	Eligible men	60% reduction in RoA	$68 per procedure	80
BCC	Heterosexual men	20% reduction in RoA	$63 pppy	100
	Low-risk women	20% reduction in RoA	$63 pppy	100
	FSW	50% reduction in RoA	$28 pppy	100
	MSM	50% reduction in RoA	$28 pppy	100
Early ART	Heterosexual men	70% reduction in RoT	$457 pppy	33
	Low-risk women	70% reduction in RoT	$457 pppy	33
	FSW	70% reduction in RoT	$457 pppy	66
	MSM	70% reduction in RoT	$457 pppy	66
PrEP	Heterosexual men	75% reduction in RoA	$95 pppy	25
	Low-risk women	75% reduction in RoA	$95 pppy	25
	FSW	75% reduction in RoA	$95 pppy	50
	MSM	75% reduction in RoA	$95 pppy	50

A unit cost is assigned to each intervention following the UNAIDS Fast Track framework [17]. For continuity with previous studies [23,24], we assume homogeneity of individual-level efficacies of early ART and PrEP with respect to risk. As this is not a forecast analysis, we do not include discounting of costs or effects, nor do we make assumptions about whether intervention costs would decrease [61] or increase [62] with scale.

VMMC, voluntary medical male circumcision; BCC, behaviour change communication; Early ART, antiretroviral therapy as prevention comprising outreach testing and offering of ART to all HIV-positive people; PrEP, pre-exposure prophylaxis; FSW, female sex workers; MSM, men who have sex with men; RoA, risk of HIV acquisition; RoT, risk of onward HIV transmission; pppy, per person per year.

Previously, we used this model to set out how prevention resources could be rebalanced across countries, and targeted by subnational epidemiology, to enable optimally cost-effective HIV prevention throughout sub-Saharan Africa [24]. Our baseline-level assumption was that ART would be provided to all HIV-positive people presenting to clinics—typically at later disease stages—regardless of gender, risk or location. This treatment-only scenario, together with an assumption of constant coverage for any historical interventions (such as past circumcision or behaviour change campaigns), comprised our counterfactual for evaluating impact. After those baseline costs were accounted for, we used the model to estimate the effect (defined as the number of infections averted during the intervention period from 2016 to 2030) and cost (approximated by unit costs multiplied by the corresponding modelled estimate of consumption) for every possible permutation of interventions and population groups in a subnational region. We then performed an optimisation analysis to maximise the local cost-effectiveness of these packages of targeted preventions, under varying sets of constraints representing policy approaches. We repeated this optimisation over a range of expenditure levels. The full model validation and resource-optimisation analysis are detailed in [24].

### Exploration of PrEP in the optimal resource distribution

Throughout this study, we focus on the role played by PrEP in the optimal allocation of prevention resources that was set out in our previous work [24]. As an example, we can consider a representative net expenditure level of $20 billion for the continent over the next 15 years (from 2016 to 2030) and examine what role PrEP plays in scale-up of the optimal prevention at this total expenditure. The $20 billion figure is obtained by assuming that current levels of HIV funding [51] will be maintained annually in the near future [21], the prevention share in HIV spending will approach 25% [57], and 90% of this prevention share will be directed to scaling up current methods (rather than to research and development of new methods). As this avoids assumptions about future domestic growth in the modelled countries and also likely declines in HIV funding by international donors, we can consider $20 billion to represent a moderate spending level for the intervention period.

**Figure 1:**
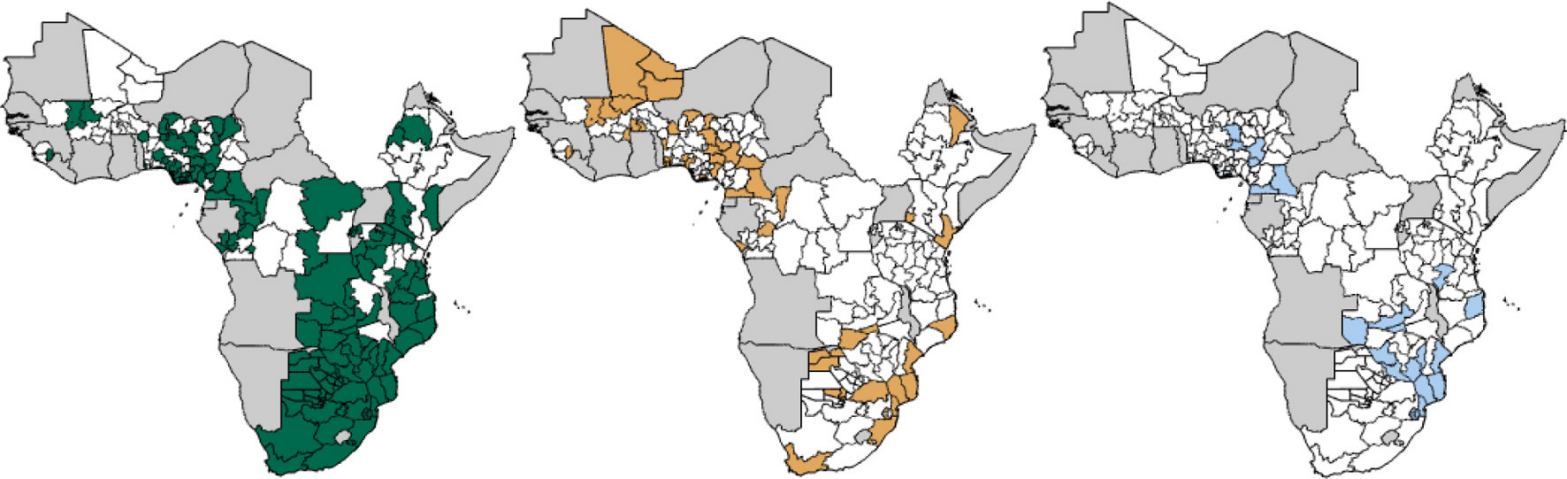
Funding of PrEP by geography and population under optimal resource allocation. Maps showing where PrEP is implemented among female sex workers (green), men who have sex with men (yellow), and the lower-risk general population (blue), or not implemented (white), for a representative total HIV/AIDS expenditure of $20 billion in the subnational regions of our modelled countries in sub-Saharan Africa. Grey areas are not modelled.

Retaining the costing and coverage assumptions from the optimisation [24] (Table 1), we can see where and in which population groups PrEP is used when the $20 billion expenditure is allocated optimally in our model (Figure 1). At this expenditure, PrEP is funded for the highest risk population groups, with FSW receiving PrEP in 63% of the modelled regions, concentrated in southern and eastern Africa where incidence and prevalence are high in this population group. MSM are also at high risk, but in the absence of detailed data, our model represents them as a more insular population and as contributing less to onward transmission than FSW. Consequently, they receive PrEP in fewer regions (29%) at this expenditure level. Additionally, the available funds allow scope for provision of PrEP to the lower incidence general population in 12% of the modelled regions.

Having illustrated the utility of our modelling framework for examining where and to whom PrEP is favoured at a given level of total prevention expenditure, we will vary this expenditure hereafter. We start from a low expenditure level at which very little prevention scale-up is possible ($1 million over the 15-year period) and increase this to a level at which all interventions can be funded in all regions ($1 trillion over the 15-year period). Over this range, we carry out a series of further analyses as follows.

Over the full range of prevention expenditures—that is, from $1 million to $1 trillion over the 15-year intervention period—we calculate the baseline HIV incidence among each population group receiving PrEP in our model. For example, at a given expenditure level, if PrEP is targeted to FSW in a subnational region, we determine what the incidence among that group would have been in 2015 in the absence of PrEP. We repeat this calculation for all groups and expenditure levels. For each expenditure level, we then determine the minimum baseline incidence over the relevant subnational regions for each group. This gives us a measure of the minimum-incidence threshold at which PrEP is implemented in the optimal allocation, as a function of overall prevention expenditure.

To examine the marginal impact of PrEP in the optimal prevention landscape, we remove PrEP from our collection of prevention interventions and repeat our optimisation analysis for the full range of total expenditure levels. This allows the three remaining prevention mechanisms (behaviour change communication, early ART, and voluntary medical male circumcision) to rebalance across locations and population groups and find a new optimal pattern in the absence of PrEP.

Finally, we consider how the prevention landscape would look if decisions regarding PrEP implementation were not optimised freely, but instead were governed by an incidence benchmark of 3 per 100 person-years, as recommended by the new WHO guidelines [16]. To do this, we determine which regions contain population groups with incidence levels of at least 3 per 100 person-years in 2016, and “force” PrEP to be funded for those population groups in those regions. Any population groups with incidence levels lower than this do not receive PrEP, even if sufficient funds are available. If funding is insufficient to provide PrEP according to the benchmark in a given region, we allow the remaining interventions to optimise in that region instead, as if PrEP were not an available option in the array of interventions. This assumes that funding is freely transferrable between prevention modalities, rather than going unused where PrEP is not affordable.

## Results

The choice of where and for whom PrEP is funded in the optimal prevention landscape depends partly on the incidence levels of the population groups. However, the full picture of the role played by PrEP is more intricate and rich than a straightforward dependence on incidence. We find that the prioritisation of PrEP also changes with the total amount of funding available for prevention and is responsive to the surrounding context of other favourable intervention modalities as determined by the local epidemiology.

**Figure 2:**
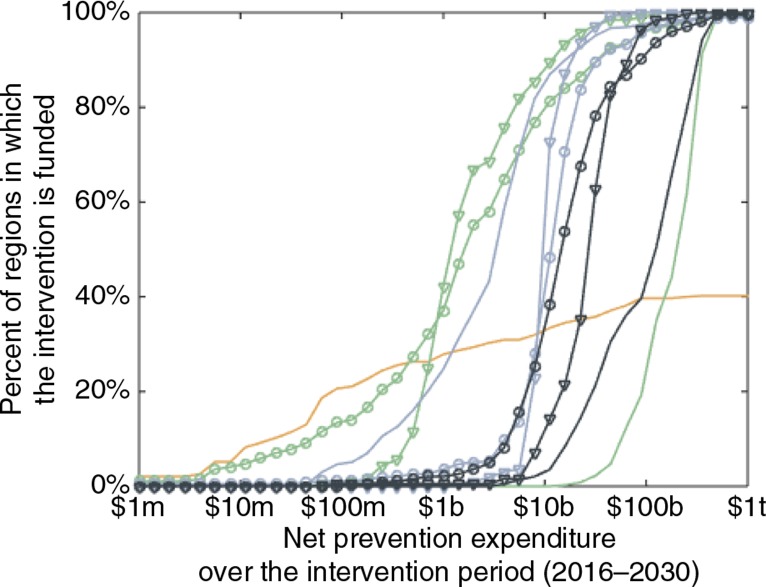
Prioritisation of PrEP among other intervention modalities. Percent of subnational (top-level administrative) regions in which PrEP (black), early ART (blue), behaviour change communication (green), and voluntary medical male circumcision (orange) are implemented among the general population (plain curves), female sex workers (circles) or men who have sex with men (triangles), over a range of net prevention expenditures for the 15-year intervention period. Female sex workers and men who have sex with men are high-risk groups, while the general population comprises low-risk women and heterosexual men. Voluntary medical male circumcision can be implemented only among uncircumcised men in regions where circumcision coverage is not already high prior to the intervention period (40% of all regions). The x-axis is on a log scale (m=million, b=billion, t=trillion).

Other interventions are more favourable than PrEP when resources are very limited (Figure 2). However, PrEP plays an increasingly significant role in the optimal landscape of combination prevention as the total expenditure increases from $6 billion onwards, under these assumptions for unit costs. As a function of expenditure, the interventions prioritised earliest are voluntary medical male circumcision and behaviour change communication for the high-risk groups, and early ART for the general population. These are followed at higher expenditures by early ART for the high-risk groups, PrEP for the high-risk groups, PrEP for the general population, and finally behaviour change communication for the general population. PrEP is prioritised earlier (with respect to expenditure) for FSW than for MSM. This is because the former group contributes more to onward transmission than the latter group in our modelling framework and thus tends to be a more cost-effective group in which to intervene.

**Figure 3:**
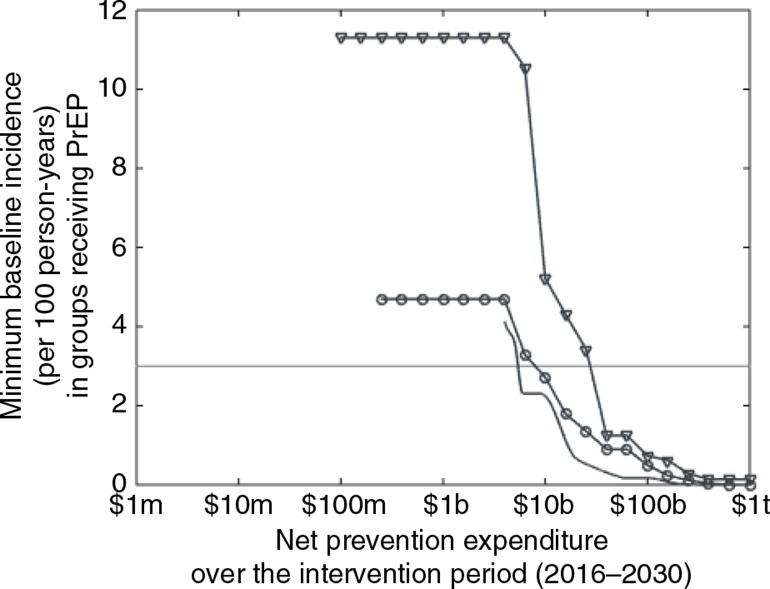
PrEP implementation by incidence. Minimum baseline incidence, or minimum incidence per 100 person-years occurring in 2015 in the absence of PrEP, in the subnational regions where PrEP is funded for female sex workers (circles), men who have sex with men (triangles), and the lower-risk general population (plain curve), as a function of net prevention expenditure over the 15-year intervention period. The horizontal line marks an incidence of 3 per 100 person-years. The x-axis is on a log scale (m=million, b=billion, t=trillion).

The baseline incidence in populations receiving PrEP is not static but decreases for all target groups as the total prevention expenditure increases (Figure 3). Until the total prevention expenditure reaches $6 billion, PrEP is prioritised to MSM only in regions where this group has a very high baseline incidence (11.3 per 100 person-years or higher) and to FSW in regions where their baseline incidence is at least 4.7 per 100 person-years. However, very few regions receive PrEP at such expenditure levels (as can be seen in Figure 2). For expenditures above $6 billion, the minimum baseline incidence levels among the high-risk populations receiving PrEP decline to zero, and these groups receive PrEP in an increasing number of regions. The general population—which comprises low-risk women and heterosexual men and has a lower average incidence than the highest risk population groups—also becomes favourable for PrEP at higher expenditures. At an expenditure of $4 billion, the optimal prevention strategy includes PrEP for the general population in regions where the baseline incidence is 4.1 per 100 person-years or higher. As with the key populations, this minimum baseline incidence declines to zero as expenditure increases. All target groups fall below the provisional WHO incidence benchmark of 3 per 100 person-years [16] at expenditures above $40 billion.

**Figure 4:**
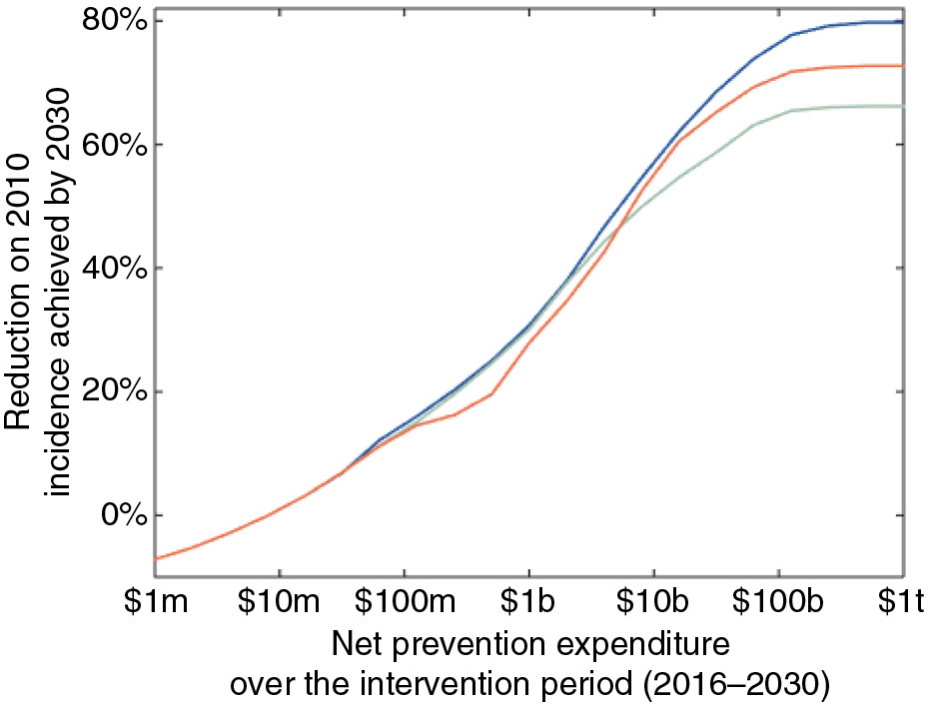
Impact of optimal prevention under different strategies for PrEP. Percent reduction on the total HIV incidence in 2010 that can be achieved by 2030, as a function of net prevention expenditure, for optimal prevention allocation with PrEP (blue), without PrEP (green) and with a benchmark incidence of 3 per 100 person-years for PrEP implementation (red). Other prevention modalities in the optimisation are behaviour change communication, early ART, and voluntary medical male circumcision. For very low prevention expenditures (<$10 million), there is so little capacity for scale-up that the epidemic rebounds slightly from its 2010 levels due to population growth. The marginal gain from including PrEP in the optimisation increases with prevention expenditure, with a maximum total reduction on 2010 incidence levels of 80%, given our assumptions around the effectiveness and coverage achievable with the interventions. The x-axis is on a log scale (m=million, b=billion, t=trillion).

If we remove PrEP from the array of available prevention modalities and optimise the implementation of the remaining modalities, we see an overall loss of impact at moderate-to-high expenditure levels (Figure 4), despite a rebalancing of the other interventions in the absence of PrEP. At expenditures below $6 billion, PrEP is used in too few places to have a significant marginal impact, but the marginal loss of impact due to removing PrEP increases with net prevention expenditure, because PrEP is most valuable at higher expenditures where it can be used in more places and population groups. At maximum—that is, for a $1 trillion expenditure—PrEP increases the impact of optimal combination prevention by 14%, and an 80% reduction on 2010 incidence is achieved by 2030, as compared to a 66% reduction without PrEP. We note diminishing returns as this maximum expenditure is approached. This is because of saturation of the populations in which it is possible to intervene, given our assumptions of imperfect effectiveness and coverage levels for the interventions (see Table 1).

If we enforce a provisional benchmark incidence of 3 per 100 person-years or higher [16] for PrEP implementation, and allow the other interventions to optimise around this, the impact pattern changes (Figure 4). At lower expenditures (between $100 million and $6 billion), a loss of impact occurs relative to both the fully optimal strategy and the strategy that would be optimal in the absence of PrEP. These two strategies are similar in this expenditure range—other interventions are more cost-effective and hence PrEP would be offered in very few places even if available—and the PrEP-benchmark scenario is worse than both. At these low expenditure levels, forcing the funding of PrEP in population groups with incidence levels that exceed the benchmark is costly and leaves little capacity for providing the other interventions that would be more favourable. Higher expenditures provide sufficient prevention capacity for the impact of the benchmark-based strategy to exceed that of the no-PrEP strategy. However, the fully optimal strategy would give PrEP to some regions in which the target population groups have incidence levels below the benchmark of 3 per 100 person-years. Without capitalising on this opportunity for extra impact, the benchmark-based strategy reduces incidence by up to 7% more than the no-PrEP strategy, in contrast to the 14% marginal gain that can be achieved by allowing PrEP to optimise freely among the other interventions.

## Discussion

Here we have considered the role of PrEP in an optimal response to the heterogeneous epidemiology of HIV, viewing it as one of several intervention modalities and taking the strategic scope of sub-Saharan Africa. We have found that an optimal prevention pattern would prioritise PrEP for high-risk key populations, particularly FSW, across sub-Saharan Africa at moderately high levels of total prevention expenditure. The lower-risk general population would also receive PrEP in some regions at higher expenditures. The choice to implement PrEP in a population group depends not only on the incidence and overall expenditure but also on the potential for impact of other possible interventions. This in turn depends upon the patterns of transmission in the population and scope for expansion of different strategies. Our analysis has shown that an optimum strategy for combination prevention without PrEP would be less effective over a range of expenditure levels than one with PrEP. Moreover, the marginal impact of PrEP would be greater at higher expenditures, achieving a reduction on 2010 incidence levels of up to 14% more than what is possible without PrEP.

The WHO guidelines provisionally define eligibility for PrEP as those with an HIV incidence meeting or exceeding the benchmark of 3 per 100 person-years in the absence of PrEP [16]. Our model finds that, under optimal allocation of total prevention funds, the minimum baseline HIV incidence among populations receiving PrEP drops as the overall expenditure increases, with all population groups receiving PrEP at incidence levels below 3 per 100 person-years at higher total expenditures. A strategy in which prevention is optimised around this PrEP benchmark incurs losses in impact for low-to-moderate expenditure levels, suggesting that if resources are thus limited, it may be preferable to forgo the offer of PrEP in favour of other interventions that are less expensive for their impact. At higher expenditure levels, the benchmark-based strategy becomes more impactful than not offering PrEP. However, it also becomes less impactful than offering PrEP in an optimal way, with losses growing considerably as the opportunity is missed to fund PrEP for population groups with incidence thresholds below the benchmark. The marginal loss reaches 7% at the highest expenditure considered here, fully half the loss that would be incurred by not implementing PrEP at all.

## Conclusions

PrEP pilot studies and demonstration projects are planned and ongoing in several countries across sub-Saharan Africa. Although many past modelling studies have estimated the impact and cost-effectiveness of PrEP as an individual intervention (reviewed in [58]), few have done so in the wider context of combination prevention and none has confronted epidemic heterogeneity both within and across multiple countries. Our study has demonstrated how PrEP can be used strategically to maximise impact with full consideration of the epidemic and financial context of sub-Saharan Africa. It is a proof-of-principle that the offer of PrEP at scale should be guided not only by incidence in the affected populations but also by total funding capacity and the context of other interventions appropriate for the local epidemics. In advocating for nuanced evaluations of PrEP and other modalities for combination prevention in resource-limited settings, this work takes an important conceptual step toward more flexible, epidemic-responsive, and effective HIV prevention decisions.

Because of data limitations, we have assumed that unit costs and effect sizes are scale invariant and the same in all countries, and that countries would not incur additional costs by sustaining a given programme in some regions and not others. Moreover, we have assumed that target populations are all equally accessible, when in reality isolated areas may be more expensive to reach than well-connected ones [59]. These factors may exaggerate the extent to which a flexible approach to programming leads to benefits. Using a finer geographical resolution than top-level subnational regions may offer further insight into the relative priority of different populations for receiving PrEP in the combination prevention landscape. For example, a microscale case study has suggested that PrEP should be prioritised first for male sex workers, then for MSM and FSW, in Nairobi (Cremin et al. Unpublished data). Stratification of the population by age may also be important, particularly for young women, who have been proposed as a priority group for PrEP implementation [60]. Differences in adherence between women aged 15 to 25 and those over 25 could, for example, raise the relative cost of reaching the younger group. If we have overestimated PrEP adherence here, then the marginal effect of PrEP has likewise been overestimated. Differences in the modelled use of PrEP among MSM and FSW are linked to our assumptions about the positions of these key populations in the sexual network and the potential for the epidemic to spread from each. As these factors are not well known in many settings, such differences should be interpreted with caution.

A number of extensions could be considered for this analysis. Further insights may be gained through exploring the sensitivity of our findings to key parameters, such as costs and levels of coverage achievable. In particular, if PrEP were to be less expensive relative to the other intervention choices, we would expect it to be implemented at lower incidence thresholds, such that it would be included in the optimal prevention bundles for more regions and population groups at lower levels of overall prevention expenditure. Conversely, if PrEP were to be more expensive at scale than what we have considered here, its role in the optimal prevention strategy could be reduced for the amount of prevention funding that is likely to be available in the future. Nevertheless, if deployed selectively with consideration of the full epidemic context, PrEP can play an important role in the optimal prevention landscape, with its full benefits to be realised if the coming years see a redoubling of financial contributions to the collective fight against HIV.
